# Adsorption–desorption nano-aptasensors: fluorescent screening assays for ochratoxin A†

**DOI:** 10.1039/d2ra00026a

**Published:** 2022-05-09

**Authors:** Velu Ranganathan, Spencer Boisjoli, Maria C. DeRosa

**Affiliations:** Department of Chemistry, Carleton University 1125 Colonel By Drive Ottawa ON K1S 5B6 Canada maria.derosa@carleton.ca +1-613-520-2600

## Abstract

In this study, a FRET-based fluorescent aptasensor for the detection of ochratoxin A (OTA) was optimized based on the quenching efficiency of single-walled carbon nanotubes (SWCNTs) and the binding affinity of aptamers. OTA aptamers were conjugated with quantum dots and adsorbed to the surface of both acid-modified and unmodified SWCNTs. The maximum fluorescence quenching efficiency of the SWCNTs were compared. Acid-modified SWCNTs (amSWCNTs) have moderate quenching efficiency, providing an optimal sensitivity for qualitative fluorescence-enhancement biosensor assays. The binding parameters of the QD-modified OTA aptamers (1.12.2 and A08min) on the surface of amSWCNTs were compared. Based on our results, the A08min aptamer is a better candidate for OTA detection. Using the A08min aptamer, the SWCNT method had a limit of detection (LOD) of 40 nM. The amSWCNT method had a significantly lower LOD of 14 nM. Turn-on fluorescent nano-aptasensors are emerging as an effective diagnostic tool for simple detection of mycotoxins. Nanocomplexes designed for the detection of mycotoxins in solution and paper-based tests have proven to be useful.

## Abstract 2

A fluorescent-enhancement biosensor was developed for the mycotoxin ochratoxin A using aptamer-modified quantum dots noncovalently immobilized on carbon nanotubes.

## Graphical abstract

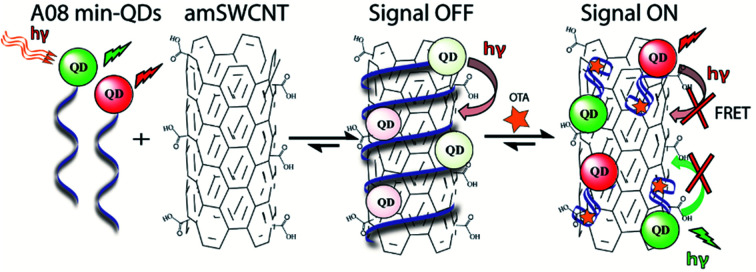

## Introduction

Single-walled carbon nanotubes (SWCNTs) have stimulated multidisciplinary interest, with potential in fields such as nano electronics,^1^ biology,^2–4^ molecular electronics, and biomedical engineering^5,6^ due to their unique mechanical, physical and chemical properties. They may also be functionalized to optimize properties for biocompatibility and biomolecular recognition.^7–9^ Specifically, one of the properties being exploited in biosensing is their quenching ability when acting as nanoquenchers and nano-scaffolds. A typical SWCNT possesses a wide absorption spectrum (approximately 500–900 nm) that overlaps with the photoluminescence spectra of various fluorophores,^10,11^ permitting Förster resonance energy transfer (FRET). During the energy transfer process, SWCNTs act as energy acceptors due to their delocalized π electrons, while fluorophores act as donors, transferring energy to ground-state SWCNTs.

Quantum Dots (QDs), also referred to as semi conducting nanocrystals, have many advantages over traditional fluorescent dyes. Among these are stronger luminescence and photostability against bleaching and physical environments such as pH, temperature, and optical turnability. Many of these properties have been exploited for immunoassays, molecular imaging, and *in vivo* biological labels.^12,13^ In addition, QDs have also been used to facilitate the development of hybrid sensing materials.^5,14^ SWCNTs can interact with QDs^15^ to cause a change in their photoluminescent properties, which is useful for *in vivo* imaging.^16,17^

Aptamers have been widely used for recognition and detection of a variety of targets in recent years. Aptamers are single stranded oligonucleotides that are selected amongst a pool of random sequences for their innate ability to recognize and bind targets with high affinity and specificity.^18^ These biosensor platforms are termed aptasensors,^19^ most of which rely on a conformational change in the aptamer between the bound and unbound state. This conformational change can be detected in a variety of ways, such as using fluorescence, polarization, energy transfer, and colour change. These functional, single stranded DNA have been found to interact non-covalently with SWCNTs.^20,21^ These complexes are stable and form by single stranded DNA (ssDNA) wrapping itself around the SWCNT through π–π stacking interactions between the nucleotide bases and the SWCNT sidewall.^20^ Once the ssDNA assembles on the surface of the SWCNT, the conjugated fluorophore's activity is quenched.

**Scheme 1:**
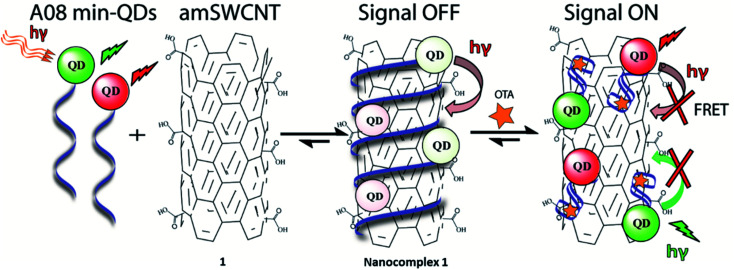
Schematic representation of A08min-QDs (CdSeTe, red spheres and CdSe/ZnS, green spheres, donors) noncovalently adsorbed on the amSWCNTs (acceptors) resulting in the fluorescence of the QDs being quenched. OTA-induced disassembly of the A08min-QDs/amSWCNTs (nanocomplex 1) leads to recovery of the QD fluorescence.

In the case of our sensor, ochratoxin A (OTA) is acting as the target analyte. OTA, one of the most abundant food-contaminating mycotoxins,^22,23^ is produced by fungi of the genera *Aspergillus* and *Penicillium*, which grow on a variety of crops. It is found in cereals and cereal-derived products as well as other commodities including coffee, cocoa, wine, and spices.^24^ OTA is a known nephrotoxin and possible carcinogen, therefore the discovery of inexpensive, widely applicable means of detection are of substantial importance. This study will outline an effective, generally simple method for the detection of OTA with the use of unmodified SWCNT (SWCNT) and acid -modified SWCNT (amSWCNT), Quantum Dots (QDs) such as CdSe/ZnS (525 nm, green-emitting) and CdSeTe (650 nm, red emitting) and the A08min OTA aptamer (A08min). In this process, A08min-QDs act as donors, while SWCNTs act as acceptors (quenchers) (See Scheme 1 for nanocomplex 1 in the presence of amSWCNTs and S1 for nanocomplex 2 in the presence of SWCNTs). The effectiveness of the nano-aptasensors was assessed by means of fluorescence, TEM, SEM imaging, and paper tests, some of which were performed under conditions mimicking field testing.

## Materials and methods

### Materials

1-Ethyl-3-(3-dimethylaminopropyl) carbodiimide hydrochloride (EDC, ≥99%), sulfo-*N*-hydroxysulfosuccinimide (sulfo-NHS, ≥98.5%), and SWCNTs were purchased from Sigma Aldrich. Ochratoxin A (OTA), Deoxynivalenol (DON), Fumonisin B1 (FB1), Patulin, Warfarin, Aflatoxin B1 and G1 from Aspergillus flavus ≥ 98.00% (HPLC), and Aflatoxins B2 and G2 ≥ 98.00% (TLC) standards were purchased from Sigma-Aldrich. Ochratoxin B (OTB) was purchased from Santa Cruz Biotechnology Canada. Carboxyl QDs 525 (Green CdSe/ZnS) and Carboxyl QD 655 (Red CdSeTe) were purchased from Life Technologies (Thermo Fisher Scientific), Burlington, ON Canada. All buffers were prepared with Millipore Milli-Q deionized water at 18 MΩ. All molecular biology grade electrophoresis chemicals were purchased from BioShop Canada (Burlington, Ontario). Reference samples (wheat, barley, corn, oats and malted barley) were provided from Trilogy Analytical laboratory. 5′NH_2_ - modified A08min and 1.12.2 aptamers were purchased from Integrated DNA Technologies (IDT).

### Instruments and software

HR-TEM images were recorded using a FEI Tecnai G2 F20 TEM, SEM images were obtained using a SEM FEG Hitachi SU-70 scanning electron microscope, UV/vis absorption spectra were obtained using a CARY 300 Bio spectrophotometer (Varian, USA) and fluorescence spectra were recorded on a fluorescence spectrophotometer (Horiba Jobin Yvon, USA) in the Dept. of Chemistry at Carleton University. *K*_D_ values were determined for the A08min aptamer by nonlinear regression analysis of the fluorescence experimental data with the one site specific binding equation using GraphPad Prism 6 software. A high-speed Sorvall legend micro 21R (thermo electron corporation) centrifuge was used for the centrifugation of solutions.

### SWCNT preparation

Unmodified SWCNTs were dissolved in MilliQ H_2_O at ∼1 mg mL^−1^ and sonicated extensively. The majority of the SWCNTs pellet out when the solution was left un-agitated for several hours; the pale-yellow supernatant was used as the unmodified SWCNT stock for trial experiments without further quantification.

### Acid-modification of SWCNTs (amSWCNTs)

Acid-modified SWCNT (amSWCNTs) were prepared using the procedure developed by B. Pan and J. Liu *et al.*^16,25^ except the mass, concentrations and filtration process were slightly modified. First, 5.2 mg of pristine SWCNTs were dissolved in 15 mL of 3.18 M HNO_3_ (diluting 5 mL of conc. HNO_3_ with 20 mL H_2_O) and placed in a 25 mL round bottom flask on a hot plate, equipped with a stir bar and condenser. The solution was refluxed for 27 hours and cooled for 3 days at room temperature. The resulting black solution was transferred to a 50 mL falcon tube and sonicated at room temperature for 60 min, before being refluxed for an additional 24 hours. After cooling, the solution was transferred back to a 50 mL falcon tube and again sonicated for 60 min at room temperature. The black solution, upon settling, separated into a yellow supernatant and a black aggregated pellet. The supernatant was passed through Spin X centrifuge filter tubes with a 0.22 μm cellulose acetate membrane (Corning) for 3 min at 2000 g. The flow-through was then passed through a 200 nm Millipore filter (1000 g, 3 min) and the amSWCNTs on the filter were washed five times (500 μL MilliQ water, 1000 g, 3 min). The amSWCNTs on the filter paper were dried in a desiccator overnight. The dried amSWCNTs were weighed and dissolved in MilliQ water to a concentration of 0.1 mg mL^−1^, then sonicated for 90 min at room temperature. After settling, the solution was a transparent grey-yellow supernatant with a small black pellet.

### Functionalization of carboxyl QDs with amine-modified aptamers

Carboxyl CdSeTe 655 QDs and CdSe/ZnS 525 core/shell QDs were conjugated with the amine-modified aptamer (A08min) using EDC and S–NHS as cross-linking reagents.^26,27^ QDs (0.93 nM, 5 μL) were mixed with 0.5 μL of EDC (25 eq. to carboxyl QDs) and 1.45 μL of S–NHS (50 eq. to carboxyl QDs) in phosphate buffered saline (PBS, pH 7.4, 450 μL). After shaking for 30 min, amine-modified aptamer (0.1 μM, 1.2 μL) was added and the solution was placed on a shaker at room temperature for an additional two hours. In this way, the amide linkage can form through the amino modification of the aptamer and the active carboxyl of the QDs. In order to remove the excess small molecules (EDC and S–NHS), the resulting samples were centrifuged at 14 000 rpm for 20 min.

### Preparation of samples for fluorescence studies

The samples of nano-complex 1 were prepared by mixing the amSWCNT (0.12 mg mL^−1^, 25 μL aqueous solution), the aptamer-modified QDs (2.5 μL of 465 pM GQDs with A08min, and 2.5 μL of 465 pM RQDs with A08min) in buffer solution (10 mM Na_2_HPO_4_, 2 mM KH_2_PO_4_, 2.7 mM KCl, and 137 mM NaCl, pH 7.4). The various concentrations of OTA were added into the above microcentrifuge tubes. These solutions were shaken and incubated for 30 min. The remaining nanocomplex samples were prepared similarly, with the following differences.

Nanocomplex 2: SWCNT (0.12 mg mL^−1^, 25 μL, aqueous solution) replaced the amSWCNTs.

Nanocomplex 3: The aptamer-functionalized QDs (930 pM GQDs, 5 μL) were modified with A08min or 1.12.2 aptamer.

Nanocomplex 4: The aptamer-functionalized QDs (930 pM RQDs, 5 μL), were prepared with A08min or 1.12.2 aptamer.

Nanocomplex 5: The aptamer-functionalized GQDs (465 pM GQDs, 2.5 μL) were prepared with A08min while the RQDs (465 pM RQDs, 2.5 μL) were prepared with 1.12.2 aptamer.

### Preparation of OTA-spiked complex extract

The extract was prepared by mixing 2 g each of sample (wheat, barley, corn, oats and malted barley) with 50 mL of deionized water and shaking for 10 min. The mixture was centrifuged for 10 min and the supernatant was filtered through Whatman grade 1 filter paper and a syringe filter (PES 0.45 μm, 30 mm diameter). The clear solution was spiked with OTA to concentrations from 1 × 10^−9^ to 1 × 10^−4^ M (each in 1 mL).

### Paper test preparation

Three rows of sample zones (∼8 mm diameter circles) were prepared on unmodified Whatman grade 41 filter paper. The top row was left empty as a control lane of OTA samples. Nanocomplexes 1 and 2 (sample 1 μL) were spotted onto the bottom two rows of sample zones and left to dry for two minutes. 1 μL of 10^−4^ to 10^−9^ M OTA solutions in complex extract were spotted on to the top and bottom rows of sample zones. The middle row of sample zones were spotted with 1 μL of the extract alone (extract control). The paper test was then illuminated with a hand-held UV light (254 nm) and visualized with a Nikon camera (model: D7000).

### TEM characterization of samples

High resolution TEM images of the A08min-amSWCNTs and A08min-SWCNTs samples were recorded by drop-casting 10 μL of a nanocomplex 1 and 2 in the absence and the presence of OTA, on a carbon-coated copper grid. Images were recorded on a FEI Tecnai G2 F20 TEM with a Schottky Field Emitter with high maximum beam current (>100 nA) electron source and imaged with a Gatan ORIUS TEM CCD Camera.

### SEM characterization of samples

Scanning electron microscope (SEM) images of the A08min-amSWCNTs and A08min-SWCNTs samples were recorded by drop-casting 50 μL of a nanocomplex 1 in the absence and the presence of OTA, on a clean gold substrate and dried at room temperature. Images were obtained using a SEM FEG Hitachi SU-70 scanning electron microscope.

## Results and discussion

### Fluorescence screening assays for OTA-aptamers with different QDs

**Scheme 2:**
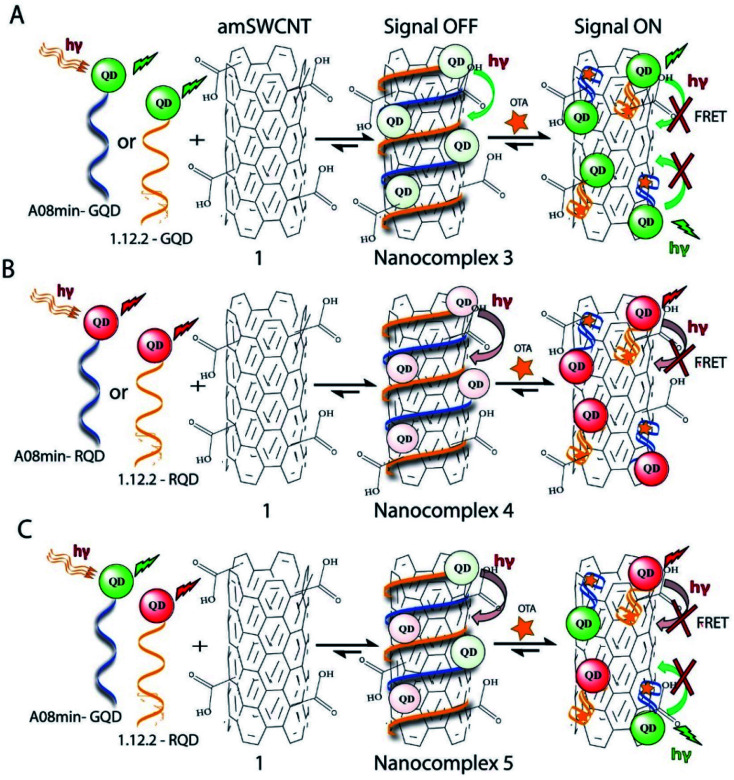
Schematic representation of A08min-QDs and 1.12.2-QDs (red sphere, CdSeTe and Green sphere, CdSe/ZnS) noncovalently wrapped on the SWCNTs. (A) Both aptamers modified with green QDs. (B) Both aptamers modified with red QDs. (C). A08min modified with green QDs and 1.12.2 modified with red QDs.

**Fig. 1:**
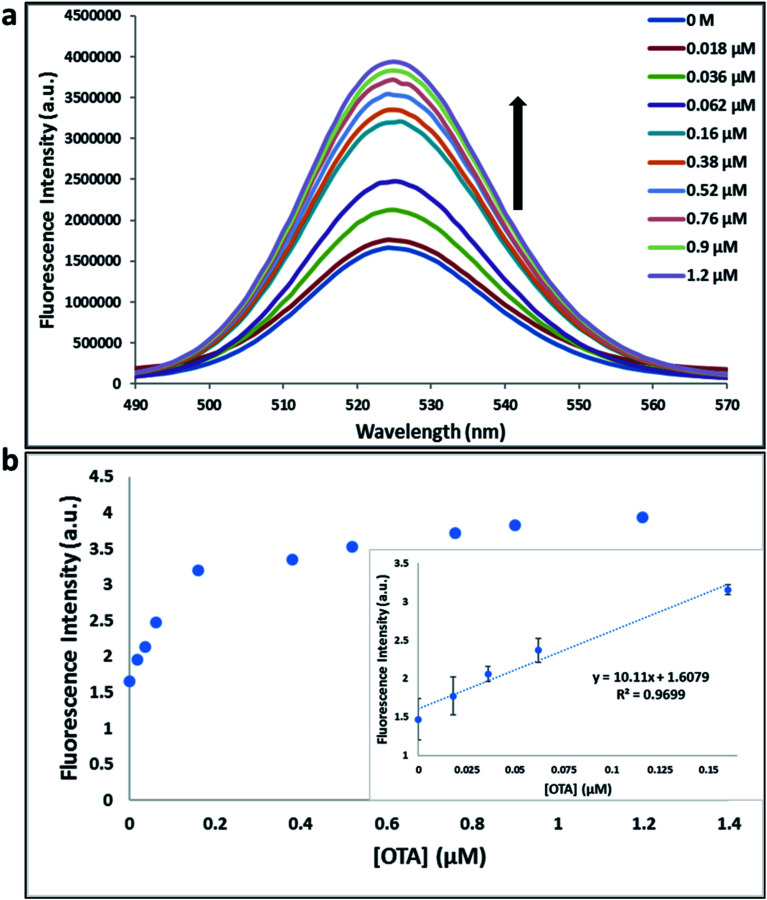
(a) The fluorescence spectra of nanocomplex 3 using A08min aptamer with increasing concentrations of OTA (0, 0.018, 0.036, 0.062, 0.16, 0.38, 0.52, 0.76, 0.9 and 1.2 μM). (b) Relative fluorescence at 525 nm (*y* = 10.11*x* + 1.6079, *R*^2^ = 0.9699) *versus* OTA concentrations. Inset: shows linear dynamic range. Triplicate experiments were performed in buffer solution (10 mM Na_2_HPO_4_, 2 mM KH_2_PO_4_, 2.7 mM KCl, and 137 mM NaCl, pH 7.4) in the presence of QDs (930 pM GQDs, 5 μL; 0.1 μM A08min, 1.2 μL) and 0.12 mg mL^−1^ amSWCNTs.

**Fig. 2:**
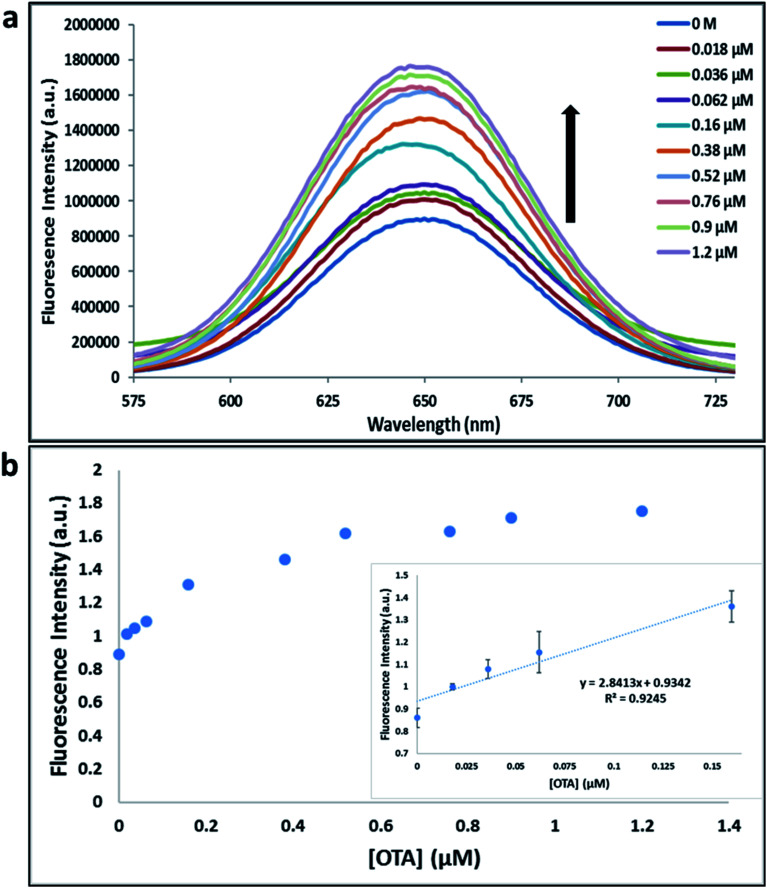
(a) The fluorescence spectra of nanocomplex 4 using A08min aptamer with increasing concentrations of OTA (0, 0.018, 0.036, 0.062, 0.16, 0.38, 0.52, 0.76, 0.9 and 1.2 μM). (b) Relative fluorescence at 650 nm (*y* = 2.8413*x* + 0.9342, *R*^2^ = 0.9245) *versus* OTA concentrations. Inset: shows linear dynamic range. Triplicate experiments were performed in buffer solution (10 mM Na_2_HPO_4_, 2 mM KH_2_PO_4_, 2.7 mM KCl, and 137 mM NaCl, pH 7.4) in the presence of QDs (930 pM RQDs, 5 μL; 0.1 μM A08min, 1.2 μL) and 0.12 mg mL^−1^ amSWCNTs.

**Fig. 3:**
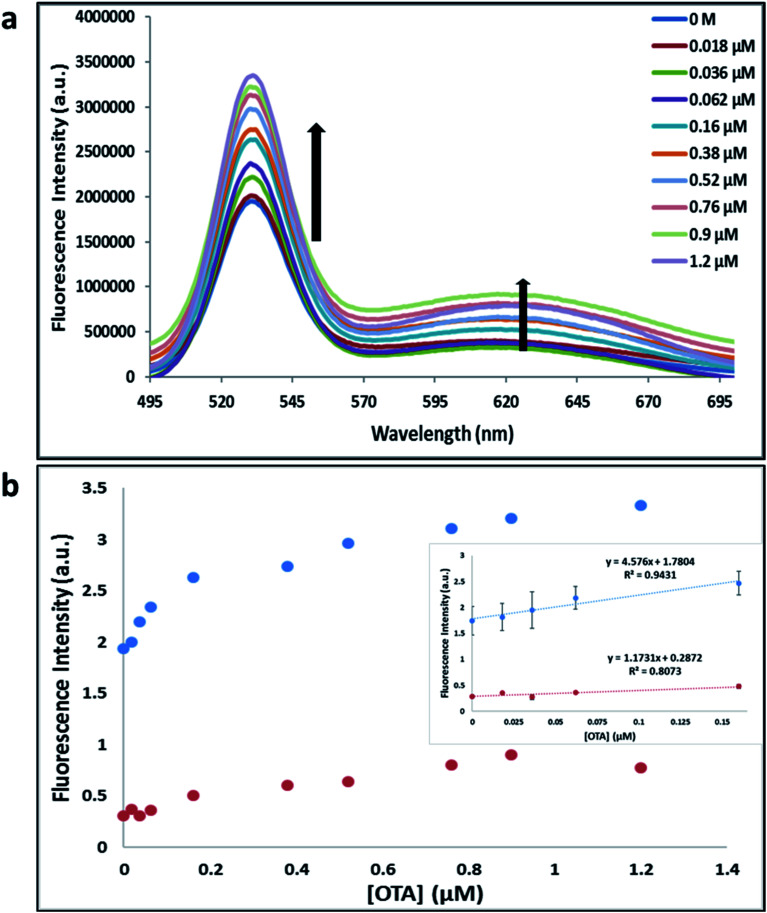
(a) The fluorescence spectra of nanocomplex 5 using A08min and 1.12.2 aptamer with increasing concentrations of OTA (0, 0.018, 0.036, 0.062, 0.16, 0.38, 0.52, 0.76, 0.9 and 1.2 μM). (b) Relative fluorescence at 532 nm (blue dots, A08min aptamer, *y* = 4.576*x* + 1.7804, *R*^2^ = 0.9431) and 630 nm (red dots, 1.12.2 aptamer, *y* = 1.1731*x* + 0.02872, *R*^2^ = 0.8073) *versus* OTA concentrations. Inset: shows linear dynamic range. Triplicate experiments were performed in buffer solution (10 mM Na_2_HPO_4_, 2 mM KH_2_PO_4_, 2.7 mM KCl, and 137 mM NaCl, pH 7.4) in the presence of QDs (465 pM GQDs, 2.5 μL with 0.06 μM A08min, 0.6 μL and 465 pM RQDs, 2.5 μL with 0.06 μM 1.12.2 aptamer, 0.6 μL) and 0.12 mg mL^−1^ amSWCNTs.

Previously determined aptamers retaining a high affinity for OTA were compared and several aptamers were selected based on their promising binding properties for the target of interest.^28–34^ Prior work contrasting various *K*_D_ detection methods in the presence of OTA, such as microscale thermophoresis (MST), fluorometric, and colorimetric assays,^28,31^ suggested that the experimental parameters play an essential role in aptamer binding affinity. The fluorescence screening experiments were performed with nanocomplexes using different OTA aptamers (A08min and 1.12.2) and QDs (red and green). First, we investigated the binding affinity of both aptamers modified with green QDs when incorporated into amSWCNTs with increasing concentrations of OTA. In two separate experiments, the GQD modified A08min and 1.12.2 aptamers were adsorbed on the amSWCNTs, resulting in the GQD fluorescence being quenched *via* FRET (scheme 2A). Upon addition of OTA to nanocomplex 3, the OTA specifically binds with its aptamer. As a result, the GQDs are released from the amSWCNTs and their fluorescence is recovered. This shows the lower limits of detection of 37 nM for A08min aptamer and 170 nM for 1.12.2 aptamer (Fig. 1a, b and S5†). Similar observations were noted in nanocomplex 4 with both aptamers modified with RQDs (scheme 2B). The LOD for A08min aptamer was 50 nM and for 1.12.2 aptamer was 174 nM under these conditions (Fig. 2a, b and S6†). In addition, we adsorbed multi-QDs (mQDs) modified aptamers (A08min-GQD and 1.12.2-RQD, Scheme 2C) to form nanocomplex 5 to give us an idea of head-to-head performance. This assay shows a LOD of 61 nM for A08min aptamer and 98 nM for 1.12.2 aptamer (Fig. 3a and b). The above experiments show that while both aptamers perform well in these experiments, the A08min aptamer produces a lower LOD when compared to 1.12.2 aptamer. The difference likely originates from the secondary and tertiary structures of the two aptamers in the bound and unbound state, as suggested from MST experiments comparing the two aptamer candidates.^28^ 1.12.2 is known to form a g-quadruplex in the presence of OTA.^34^ The limited structural change that results from the formation of the OTA-1.12.2 complex may be responsible for the slightly elevated LOD. Thus, the A08min aptamer is a better candidate for OTA detection. The apparent *K*_D_ values in these complex systems were determined for the A08min aptamer obtained using the fluorescence experimental data through nonlinear regression analysis. The A08min aptamer apparent *K*_D_s were different in different environments, which was anticipated as there would be variable affects of the interaction of the aptamer with the surface that could impact the measured affinity. The aptamer binds with high affinity (nanomolar range from 18–54 nM) in solutions containing amSWCNT (Fig. S10†). Moreover, these data are in good agreement with our previous reported analysis. The microscale thermophoresis (MST) showed *K*_D_ = 97 ± 33 nM, the fluorometric SYBR Green assay showed *K*_D_ = 169 ± 52 nM and affinity chromatography assay (magnetic beads) showed *K*_D_ = 406 ± 166 nM.^31^ These results confirm that the A08min aptamer binds OTA with excellent affinity and therefore may be the best aptamer for our OTA assays.

### Fluorescence solution studies

With the optimal OTA aptamer selected, we retained the concept of dual color detection (employing both the GQD and RQD labels with the A08 aptamer) for two reasons. Firstly, having two signals to monitor could allow for an internal check to confirm binding results. Secondly, this format could be adapted to multiplexing in future experiments. The QDs employed for this work have two strong emission wavelengths, one at 525 nm (GQD) corresponding to CdSe/ZnS and the other at 650 nm (RQD) corresponding to CdSeTe (Fig. S3a†). The emission spectra of GQD and RQD overlap with the absorption of amSWCNTs (Fig. S3b†) allow FRET to occur, resulting in QDs fluorescence quenching. The emission spectra of A08min-QDs in the presence of different concentrations of SWCNT and amSWCNTs were investigated. As shown in Fig. S4a and b,† the fluorescence intensities of A08min-QDs decrease while the concentration of amSWCNTs ranging from 0.0 to 0.12 mg mL^−1^, was increased. The maximum quenching was observed at an amSWCNT concentration of 0.12 mg mL^−1^. The GQD fluorescence was quenched to 37% of maximum. Similarly, the RQD fluorescence was quenched to 13% of maximum. The interaction holding the A08min-QDs to the amSWCNTs is noncovalent and yields nanocomplex 1. It forms through the π–π stacking/van der Waals forces of the nucleotide bases to the amSWCNT sidewall,^35–37^ bringing the QDs in close proximity to the amSWCNT. The energy transfer occurs from the aptamer-QDs, which act as donors, to the amSWCNTs, which act as acceptors, resulting in the fluorescence quenching of A08min-QDs. The maximum fluorescence quenching efficiency, observed when 0.12 mg mL^−1^ of each amSWCNT was added, was greater than 65% for amSWCNT, and 99% for SWCNT (nanocomplex 2). In a recent study of the noncovalent interactions of SWCNTs in the presence of fluorescein derivatives, quenching efficiency was between 67% and 98%.^38,39^ This limited quenching efficiency of amSWCNTs provides sufficient sensitivity for fluorescence enhancement qualitative biosensor assays.

**Fig. 4:**
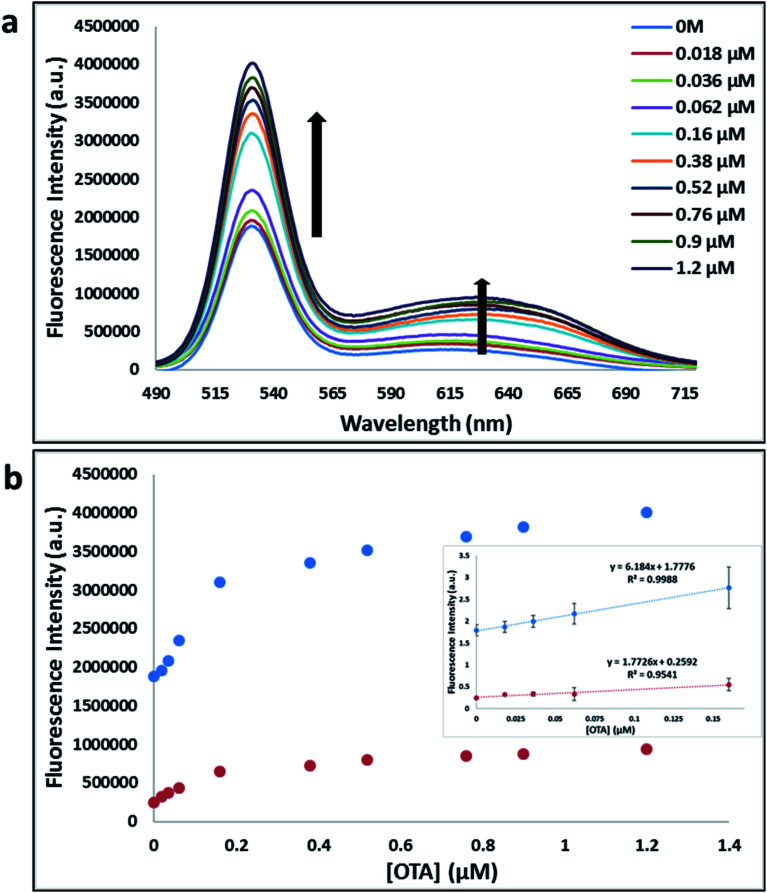
(a) The fluorescence spectra of nanocomplex 1 with increasing concentrations of OTA (0, 0.018, 0.036, 0.062, 0.16, 0.38, 0.52, 0.76, 0.9 and 1.2 μM). (b) Relative fluorescence at 530 nm (blue dots, *y* = 6.184*x* + 1.7776, *R*^2^ = 0.9988) and 630 nm (red dots, *y* = 1.7726*x* + 0.02592, *R*^2^ = 0.9542) *versus* OTA concentrations. Inset: shows linear dynamic range. Triplicate experiments were performed in buffer solution (10 mM Na_2_HPO_4_, 2 mM KH_2_PO_4_, 2.7 mM KCl, and 137 mM NaCl, pH 7.4) in the presence of QDs (465 pM GQDs, 2.5 μL; 0.06 μM A08min, 0.6 μL and 465 pM RQDs, 2.5 μL; 0.06 μM A08min 0.6 μL) and 0.12 mg mL^−1^ amSWCNTs.

**Fig. 5:**
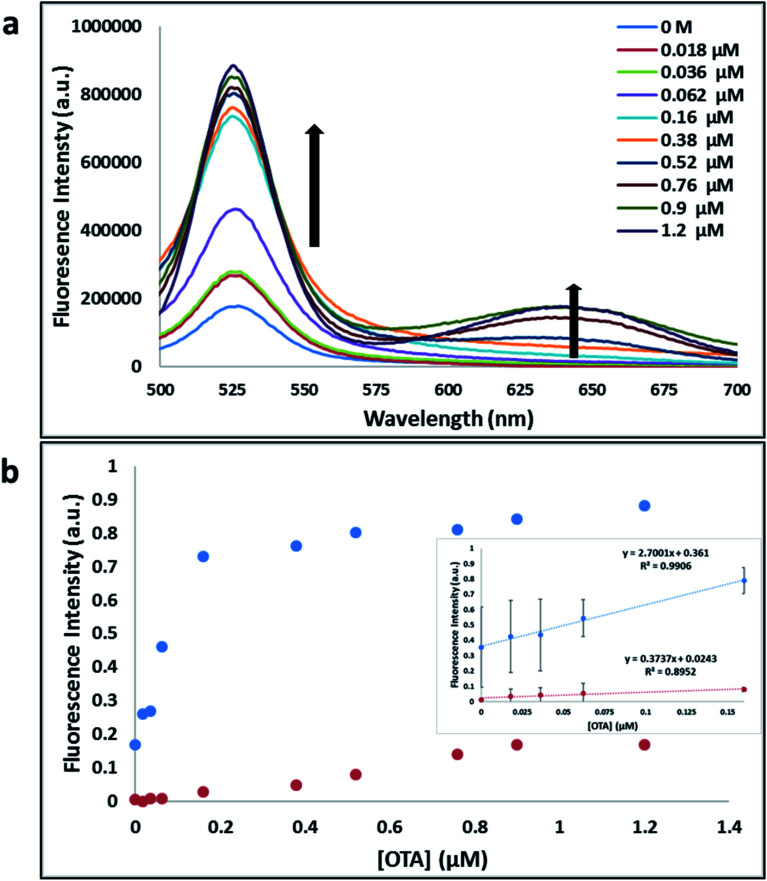
(a) The fluorescence spectra of nanocomplex 2 with increasing concentrations of OTA (0, 0.018, 0.036, 0.062, 0.16, 0.38, 0.52, 0.76, 0.9 and 1.2 μM). (b) Relative fluorescence at 530 nm (blue dots, *y* = 2.7001*x* + 0.361, *R*^2^ = 0.9906) and 645 nm (red dots, *y* = 0.3737*x* + 0.0243, *R*^2^ = 0.8952) *versus* OTA concentrations. Inset: shows linear dynamic range. Triplicate experiments were performed in buffer solution (10 mM Na_2_HPO_4_, 2 mM KH_2_PO_4_, 2.7 mM KCl, and 137 mM NaCl, pH 7.4) in the presence of QDs (465 pM GQDs, 2.5 μL; 0.06 μM A08min, 0.6 μL and 465 pM RQDs, 2.5 μL; 0.06 μM A08min 0.6 μL) and 0.12 mg mL^−1^ SWCNTs.

**Fig. 6:**
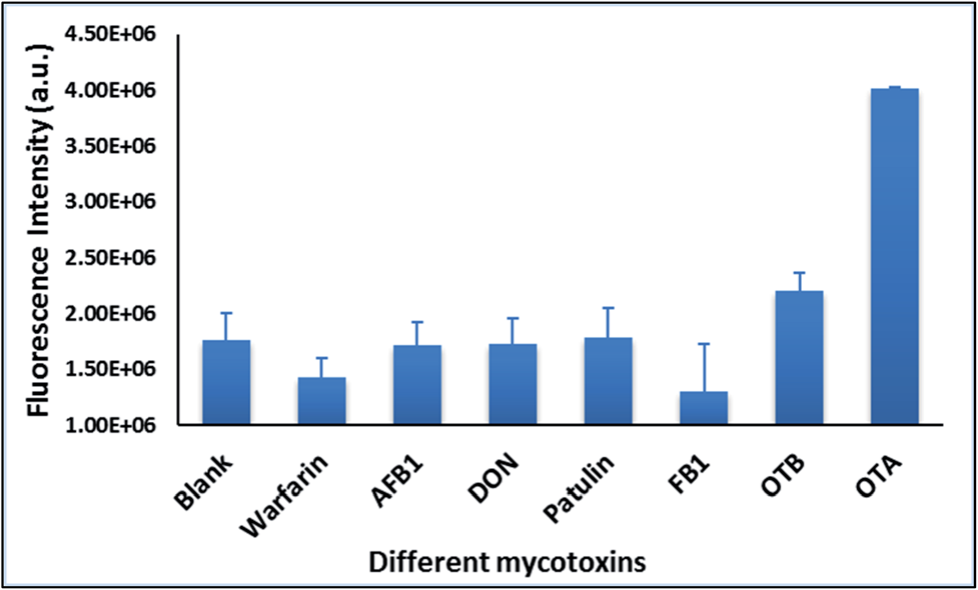
The selectivity experiments of nanocomplex 1 for OTA over other mycotoxin standards such as Warfarin, AFB1, DON, Patulin, FB1 and OTB were performed. Fluorescence intensity at 530 nm is plotted for all the mycotoxin at 1.5 μM. Triplicate experiments were performed for each mycotoxin standard.

With the addition of OTA (concentration range of 0 M–1.2 μM) to nanocomplex 1, OTA binds specifically with its aptamer, leading to a conformational change of the A08min aptamer. This conformational change results in the disruption of the π–π stacking interactions between A08min-QD and the amSWCNT, thus causing it to release from the amSWCNT surface. As a result, FRET is inhibited and the fluorescence of A08min-QDs is recovered (Scheme 1 and Fig. 4a). This assay showed good linearity in the range from 0 M to 160 nM OTA with a limit of detection (using 3.3 × Syx/slope) of 14 nM (Fig. 4b). Similar experiments were performed for OTA with nanocomplex 2. The experiments were carried out using the same concentrations and conditions listed above which were used to perform the nanocomplex 1 method. Similar observations were noted to those for detection of OTA with nanocomplex 1. This fluorescence assay showed good linearity in the range from 0 M to 160 nM OTA with a limit of detection (using 3.3 × Syx/slope) of 40 nM (Fig. 5a, b and S1†). The selectivity experiments for OTA over other mycotoxin standards such as Warfarin, AFB1, DON, Patulin, FB1 and OTB were added to the nanocomplexes under the same experimental conditions. The results demonstrated that no significant change in the fluorescence intensity occurred (Fig. 6). These observations indicate that nanocomplex 1 is selective for OTA.

### HR TEM studies

**Fig. 7:**
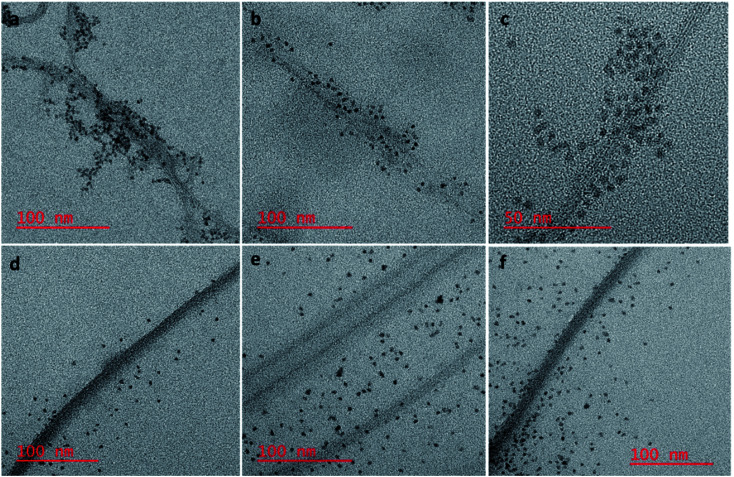
HR-TEM images of nanocomplex 1 (mixture of A08min-mQDs and amSWCNT), where the QDs are closely associating with the amSWCNT side wall (a–c). Nanocomplex 1 after the addition of 1.2 μM of OTA; OTA specific binding with its aptamer results in dispersal of the QDs from the surface (d–f).

**Fig. 8:**
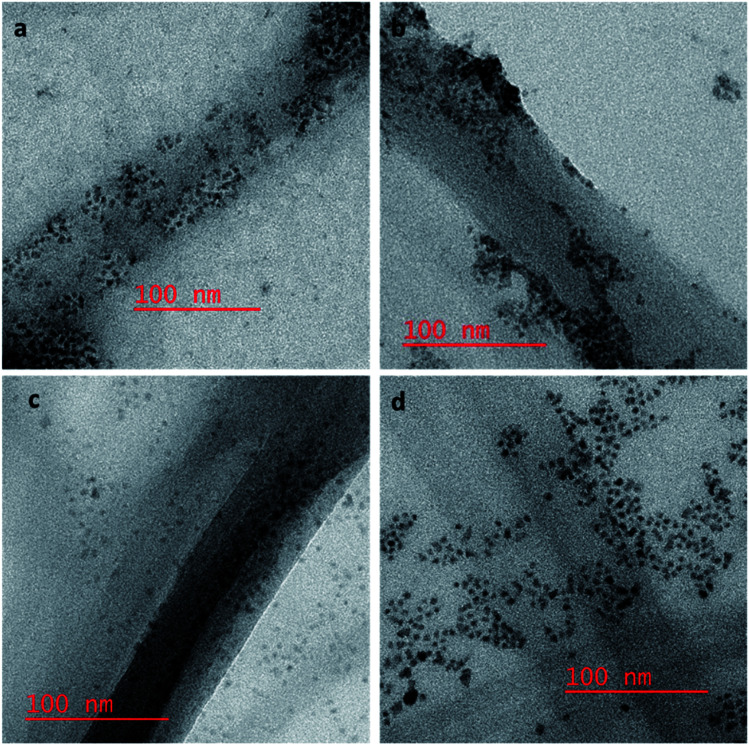
HR-TEM images of nanocomplex 2 are the mixture of A08min-mQDs and SWCNT, where the QDs are closely associating with the SWCNT side wall (a and b). Nanocomplex 2 after the addition of 1.2 μM of OTA, OTA specific binding with its aptamer results in dispersal of the QDs from the surface (c and d).

To confirm our hypothesis that the displacement of the aptamer from the SWCNT surface is what is leading to our signal enhancement, TEM samples were prepared by drop casting the solutions onto a carbon coated copper grid. Bundles of amSWCNT that are 40–100 nm in diameter and amSWCNT bundles at 20–50 nm in diameter could be observed by TEM. CdSe/ZnS and CdSeTe QDs were also imaged; the average diameter of each was 3.5 nm and 5.0 nm respectively (Fig. S2†). The proposed mechanism for the assembly and disassembly of nanocomplex 1 and nanocomplex 2 was supported by TEM analysis. Fig. 7a–c shows A08min-QDs (dark particles) on the outer surface of amSWCNT (tube shaped structure). This indicates the A08min-QDs were adsorbed onto the amSWCNT through the noncovalent π–π stacking interactions to form nanocomplex 1. Fig. 7d–f shows the separation of A08min-QDs from the amSWCNT upon addition of OTA. This indicates that A08min-QD preferably binds OTA over the amSWCNT. The same experimental conditions were used for nanocomplex 2 and similar results were observed. TEM images of nanocomplex 2 in the absence and the presence of OTA are illustrated in Fig. 8. Nanocomplexes 1 and 2 assembly and disassembly were also observed throughout the sample area in the grid, and the images are from multiple regions as presented in ESI (Fig S7 and S8)†.

### SEM studies

**Fig. 9:**
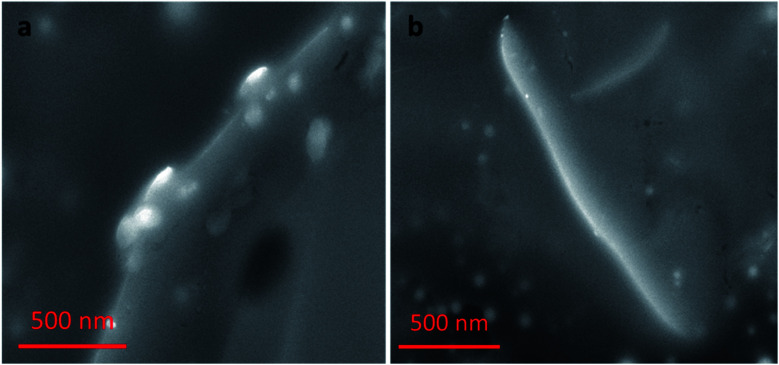
(a) SEM images of nanocomplex 1 is the mixture of A08min-QDs and amSWCNT, where the QDs are closely associating with the amSWCNT side wall. (b) Nanocomplex 1 after the addition of 1.2 μM of OTA, OTA specific binding with its aptamer and resulting in dissociation of the QDs.

Nanocomplex 1 was also characterized by SEM. SEM samples were prepared by solvent evaporation on a gold planar surface. All of the SEM studies discussed follow the same experimental conditions as the previous TEM study. The images of nanocomplex 1 illustrate assembly in the absence, and disassembly in the presence of OTA. Fig. 9a shows nanocomplex 1 in the absence of OTA, where the A08min-QDs adsorb to the surface of the amSWCNT. Upon the addition of OTA, OTA specific binding with its aptamer results in the disassembly of the A08min-QDs and amSWCNTs from nanocomplex 1. This is depicted in Fig. 9b where the A08min-QDs and amSWCNTs are well-dispersed.

### OTA detection in extracts

**Fig. 10:**
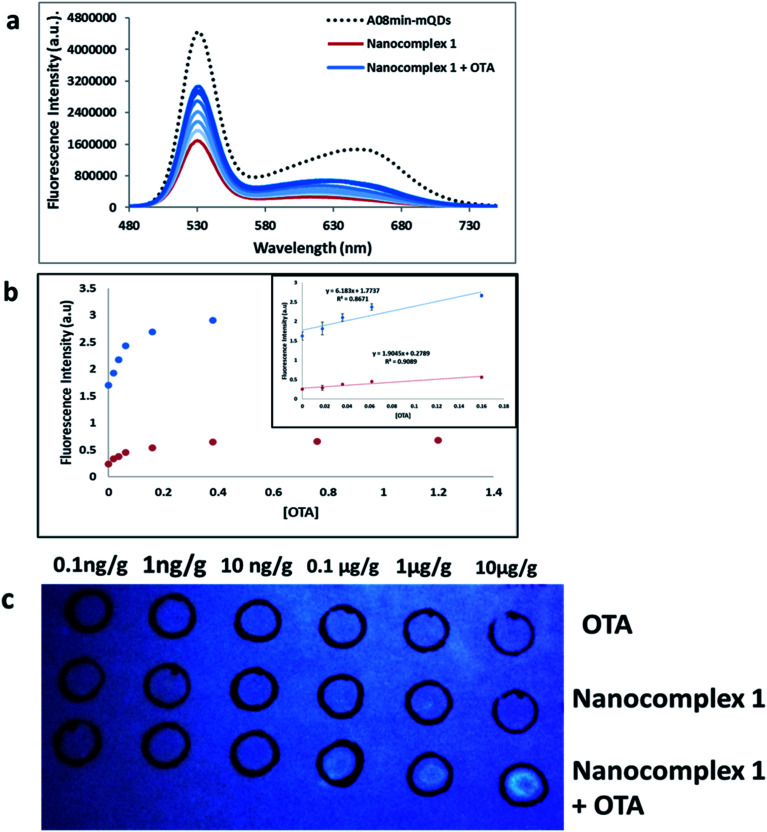
(a) The fluorescence spectra of nanocomplex 1 with OTA-spiked extracts. After assembly of the nanocomplex 1 with A08 aptamer (red line), OTA-induced disassembly leads to an increase in the fluorescence signals (blue lines), to 55% of the original signal prior to assembly (dashed line). The loss of fluorescence in comparison to buffer solution is attributed to matrix effects. (b) Relative fluorescence at 532 nm (blue dots, *y* = 6.1863*x* + 1.7737, *R*^2^ = 0.8671) and 630 nm (red dots, *y* = 1.9045*x* + 0.2789, *R*^2^ = 0.9089) *versus* OTA concentrations. Inset: shows linear dynamic range. (c) 1 μL OTA from 10^−4^ to 10^−9^ M was spiked into complex extract and spotted into top row as a control. Nanocomplex 1 sample (1 mL) was spotted into middle row as a control. Both OTA-spiked extracts from 10^−4^ to 10^−9^ M and nanocomplex 1 were spotted onto the bottom row (Table 1).

Additionally, other tests were performed using OTA-spiked extracts to test the effectiveness of this solution-based sensing. These extracts were a mixture of wheat, barley, corn, oat and malted barley to simulate authentic testing conditions. QD fluorescence was recovered to roughly 55% of the original signal intensity, slightly less than nanocomplex 1 in buffer demonstrated in the previous experiments (Fig. 10a). This assay showed good linearity in the range from 0 M to 160 nM OTA with a limit of detection (using 3.3 × Syx/slope) of 0.018 μg g^−1^ (Fig. 10b). This slight difference in quenching effect is likely due to other components of the extract contributing as quenchers to the QDs. Still, the nanocomplex 1 approach was deemed feasible for mycotoxin detection in both simple and complex matrices.

Furthermore, paper-based detection systems were developed based on the approach of nanocomplex 1. Paper assays are gaining widespread attention in biosensor development due to their low cost and simplicity. DNA-nanoparticle paper-based assays have been developed for the detection of nucleic acid and non-nucleic-acid targets.^40–42^ To set up the paper-based test, first OTA spiked extracts were dabbed onto the top and bottom rows of the filter paper at concentrations ranging from 10 pg g^−1^ to 10 μg g^−1^. The top row was used as a control, while nanocomplex 1 was added to the bottom row. Nanocomplex 1 was also spotted on the middle row as a control. When compared to controls, visual detection of OTA, down to 100 ng g^−1^, could be observed with a simple hand-held UV-light and camera, without paper modification or the need for expensive equipment (Fig. 10c). When attempting the paper-based test for extracts with nanocomplex 2, using similar experimental conditions described previously, the results were less successful. Only in the most concentrated trial, at a concentration of 100 μM, was any fluorescence established (Fig. S9†). This clearly establishes the superiority of nanocomplex 1 for the sensing of OTA in extracts as the presence of amSWCNTs led to the 100-fold increase in sensitivity over nanocomplex 2.

For comparison purposes, we reviewed several FRET-based aptasensors for OTA which use different recognition materials such as Graphene oxide (GO) and SWCNTs (Table 1). These methods were less sensitive, having LODs from 17.2 nM–24.1 nM^37–39,41–44^ compared to our FRET method which had LOD of 14 nM. In addition, our method has several advantages over FRET-based OTA sensors. It is relatively fast, requiring only 30 min to perform the experiment. Additionally, we compared the sensitivity and selectivity for both unmodified and modified SWCNTs and two different OTA aptamers labeled with two different QDs for easy fluorescence interpretation. In these regards, our method is better than other detection methods. Our proposed amSWCNT aptasensor was simple, providing better reproducibility and convenient observations within 30 min. It was successfully applied in paper tests and for real sample analysis. This method is promising for future on-site mycotoxin testing applications.

**Table 1:** Comparison of the LODs of several FRET based aptasensors for OTA detection

Detection technique	Aptasensors principle	LOD	Ref.
Fluorescence	FRET based aptasensor for turn-on method using carbon nanotubes and FAM	24.1 nM	37
Fluorescence	FRET based aptasensor for turn-on methods using FAM, PVP-coated graphene oxide and graphene oxide (GO)	21.8 nM	38
		1.9 μM	38
Fluorescence	FRET based aptasensor method using FAM and AuNPs	22.7 nM	39
Fluorescence	FRET based aptasensor method using SWCNHs and FAM	17.2 nM	43
Fluorescence	FRET based aptasensor assay using nano-graphite and FAM	20 nM	44
Fluorescence	FRET based aptasensor using SWCNT, carboxyl modified SWCNT and multi-QDs	40 nM	This work
		14 nM	

## Conclusions

The results confirm that nanocomplex 1 effectively translates the binding of the aptamer to OTA into a specific biosensor. This binding was used as a switch, turning on the fluorescence in the presence of OTA. This approach proved to be effective in both paper and solution-based tests, even when the target was introduced in agricultural extracts. Multi QDs (mQDs) were used to emit different wavelengths of light upon excitation with a UV lamp. Future applications may manipulate this property to create a multi-toxin biosensor by conjugating different coloured QD to aptamers selected for different toxins.

## Conflicts of interest

There are no conflicts to declare.

## Supplementary Material

RA-012-D2RA00026A-s001
