# Supplementary material for: Adsorption–desorption nano-aptasensors: fluorescent screening assays for ochratoxin A
Source: RSC Adv. 2022 May 9;12(22):13727–39. doi: 10.1039/d2ra00026a (PMC9081825; doi:10.1039/d2ra00026a)
Supplement: RA-012-D2RA00026A-s001 [file RA-012-D2RA00026A-s001.pdf]

Electronic Supplementary Material (ESI) for RSC Advances.
This journal is © The Royal Society of Chemistry 2022

# Supporting Information

## Adsorption–Desorption Nano-Aptasensors: fluorescent screening assays for Ochratoxin A

Velu Ranganathan, Spencer Boisjoli and Maria C. DeRosa*

Department of Chemistry, Carleton University, 1125 Colonel By Drive, Ottawa, ON K1S 5B6, Canada.
*Correspondence: maria.derosa@carleton.ca (M.C.D.); Tel: +1-613-520-2600 (ext. 3844) (M.C.D.).

Table of Contents:

I. Synthetic Procedures
   a. Aptamer sequences and buffer

II. Figures
   a. S1- Schematic representation of A08min-QDs and SWCNTs
   b. S2- TEM and SEM images of SWCNT and amSWCNT and mQDs
   c. S3- Absorption and emission spectra of QDs, emission spectra of QDs overlap with the absorption spectrum of amSWCNT
   d. S4- Fluorescence quenching of A08min-QDs with SWCNTs and amSWCNT
   e. S5- Fluorescence of nanocomplex **3** (using 1.12.2-GQD) with and without OTA
   f. S6- Fluorescence of nanocomplex **4** (using 1.12.2-RQD) with and without OTA
   g. S7- TEM images of nanocomplex **1** with and without OTA
   h. S8- TEM images of nanocomplex **2** with and without OTA
   i. S9- Paper test image for nanocomplex **2** with OTA spiked
   j. S10- $K_D$ values determined for the A08min aptamer obtained using the fluorescence experimental data

# I. Synthetic Procedures

### a) Aptamer sequences and buffer

| OTA aptamers | Sequence 5' to 3' |
|---|---|
| A08min | 5’-$H_2N$-GGC AGT G TG GGC GAA TCT ATG CGT ACC GTT CGA TAT CGT G-3’ |
| 1.12.2 | 5’ $H_2N$-GAT CGG GTG TGG GTG GCG TAA AGG GAG CAT CGG ACA-3’ |
| | **Buffer** |
| A08min | 10 mM $Na_2HPO_4$, 2 mM $KH_2PO_4$, 2.7 mM KCl, and 137 mM NaCl (pH 7.4) |
| 1.12.2 | 10 mM HEPES, 120 mM NaCl, 5 mM KCl, 10 mM $CaCl_2$ (pH 7.0) |

## II. Figures

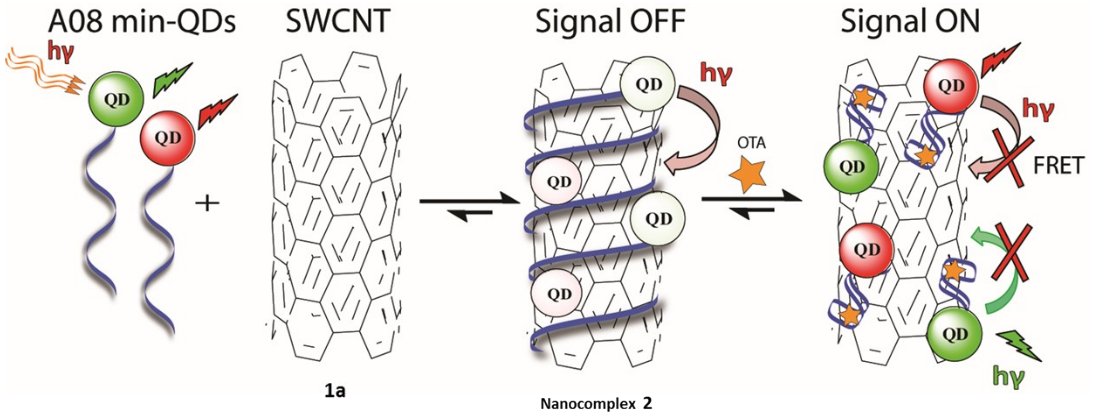


Fig. S1**.** Schematic representation of A08min-QDs (CdSeTe, red spheres and CdSe/ZnS, green spheres, donors) noncovalently wrapped on the unmodified SWCNTs (acceptors) resulting in the fluorescence of the QDs being quenched. OTA-induced disassembly of the A08min-QDs/SWCNTs (nanocomplexes) leads to recovery of the QD fluorescence.

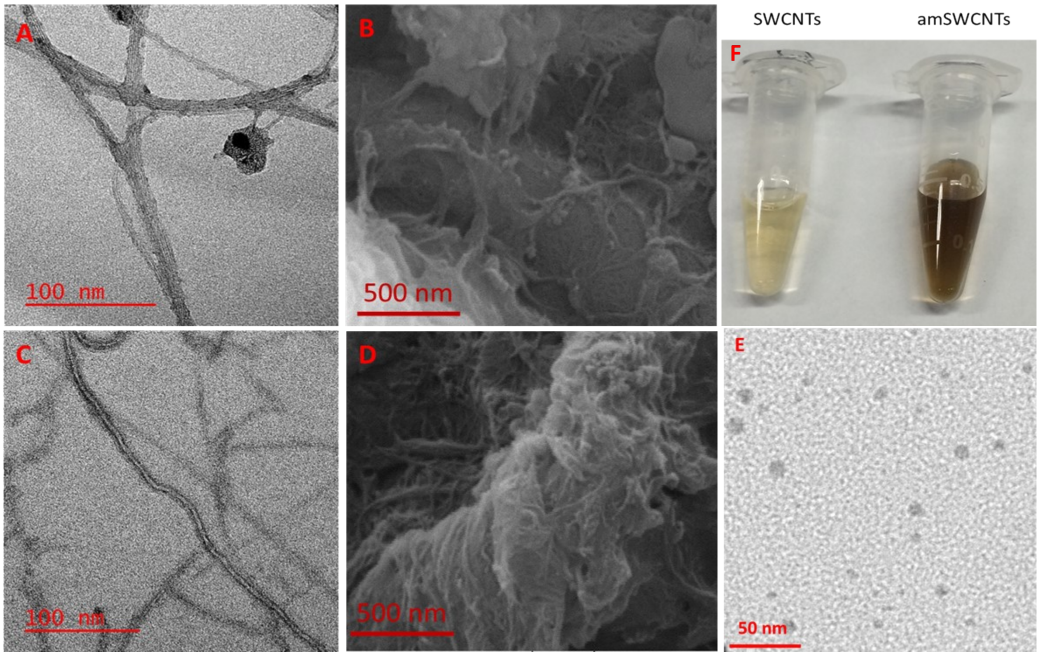


Fig. S2. (A & C). TEM images of SWCNT and amSWCNT. The presence of SWCNT bundles, 30-60 nm in diameter and amSWCNT bundles, 20-40 nm in diameter. (B & D) SEM images of SWCNT and amSWCNT. (E) TEM image of multi quantum dots (mQDs) (CdSe/ZnS, average size 3.5nm and CdSeTe, average size 5.0 nm). TEM and SEM samples were prepared with 0.93 nM mQDs and 1 mg/mL SWCNTs. (F) Photo images of SWCNT and amSWCNT in water.

a
b

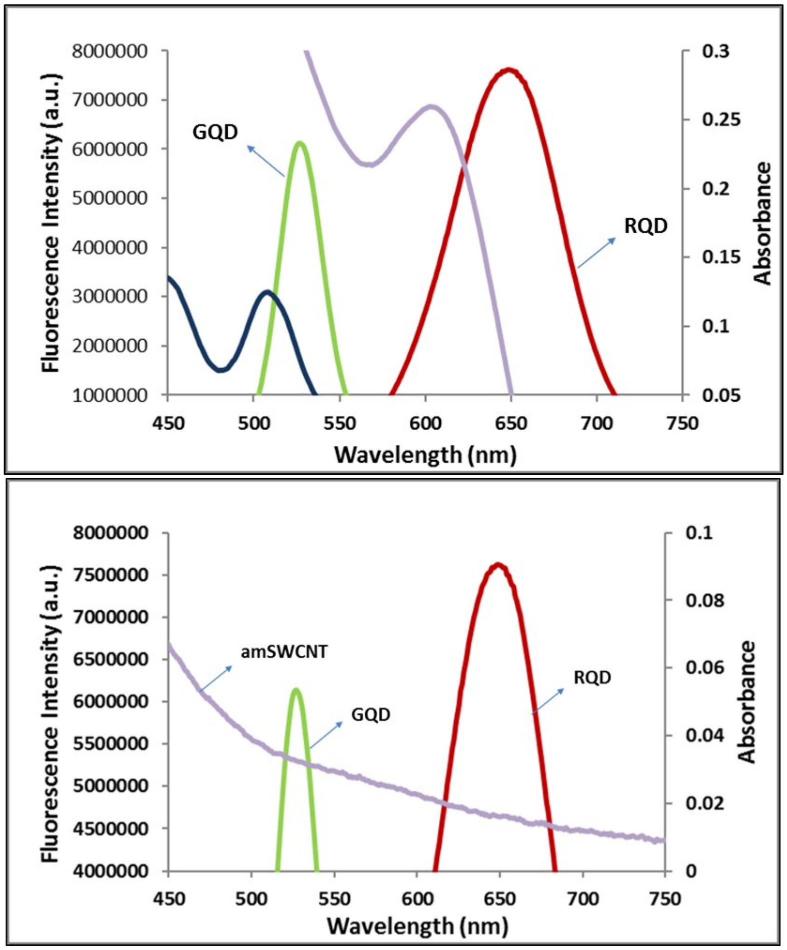


Fig. S3. a). Absorption (blue and purple lines) and emission spectra (green and red lines) CdSe/ZnS and CdSeTe (QDs). b) Emission spectra (green and red lines) of QDs overlaps with the absorption spectrum of amSWCNTs. All experiments were performed in buffer solution (10 mM $Na_2HPO_4$, 2 mM $KH_2PO_4$, 2.7 mM KCl, and 137 mM NaCl, pH 7.4)

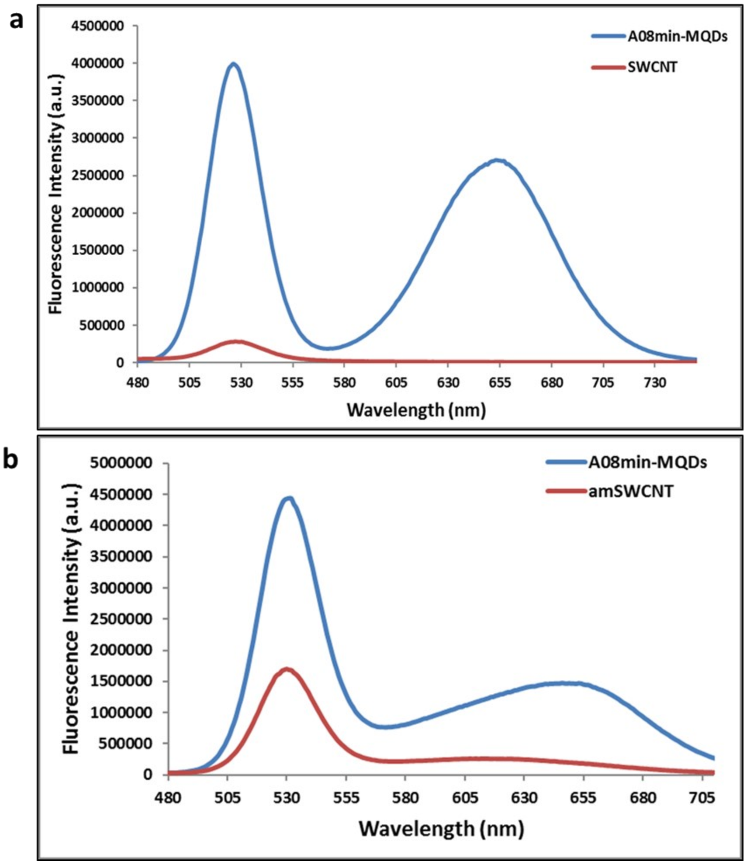


Fig. S4. The fluorescence quenching of A08min-QDs (blue line) in the presence of a). SWCNTs (red line) and b). amSWCNTs (red line). SWCNT and amSWCNT concentrations ranged from 0 to 0.12 mg/mL.

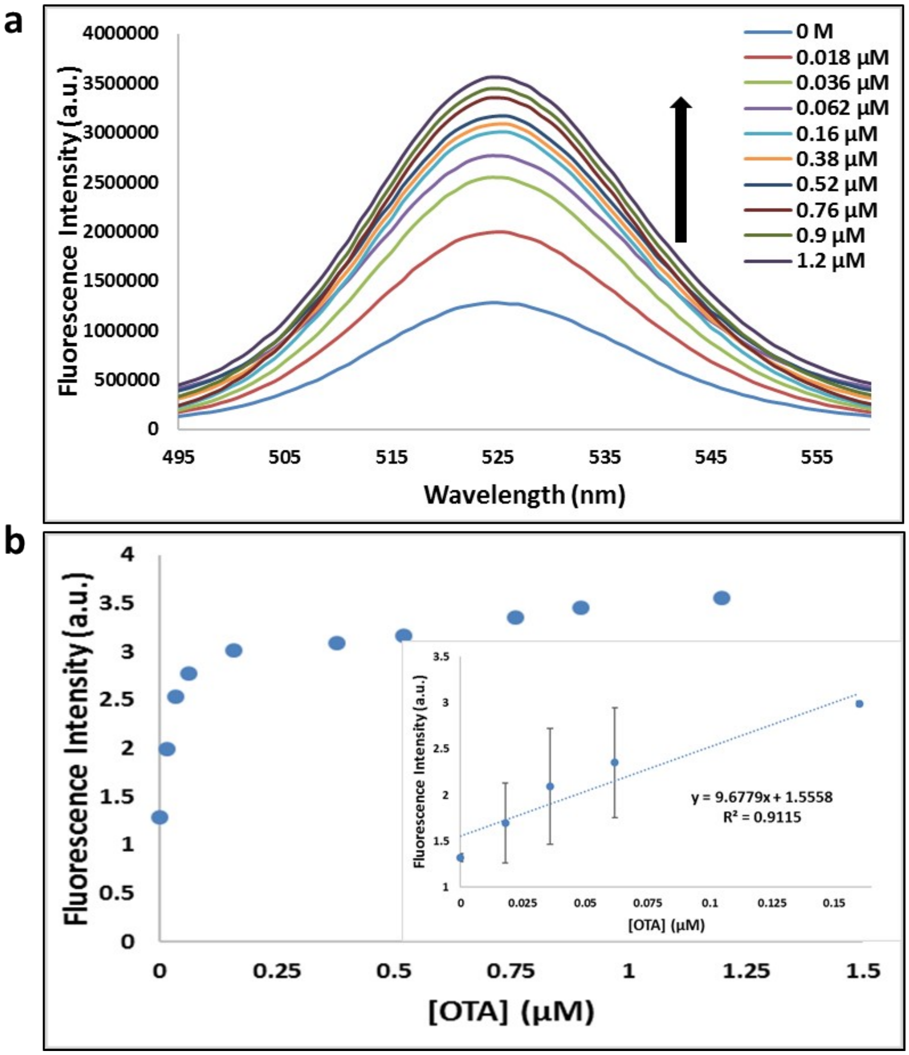


Fig. S5. a). Fluorescence recovery with increasing OTA (0, 0.018, 0.036, 0.062, 0.16, 0.38, 0.52, 0.76, 0.9 and 1.2 µM). b). Relative fluorescence at 525 nm (y = 9.6779x + 1.5558, $R^2$ = 0.9115) versus OTA concentrations. Inset: shows linear dynamic range. Triplicate experiments were performed in buffer solution (10 mM $Na_2HPO_4$, 2 mM $KH_2PO_4$, 2.7 mM KCl, and 137 mM NaCl, pH 7.4) in the presence of 0.1 µM 1.12.2 aptamer, 0.93 nM GQDs and 0.12 mg/mL amSWCNTs.

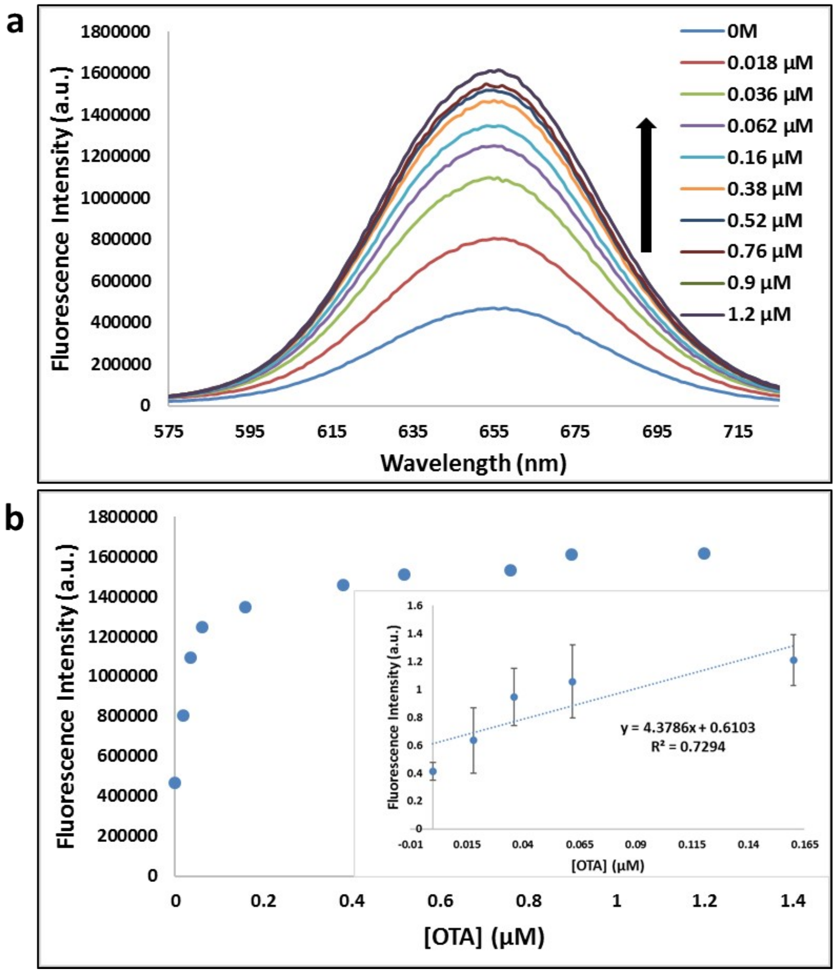


Fig. S6. a). Fluorescence recovery with increasing OTA (0, 0.018, 0.036, 0.062, 0.16, 0.38, 0.52, 0.76, 0.9 and 1.2 µM). b). Relative fluorescence at 650 nm (y = 4.3786x + 0.6103, $R^2$ = 0.7294) versus OTA concentrations. Inset: shows linear dynamic range. Triplicate experiments were performed in buffer solution (10 mM $Na_2HPO_4$, 2 mM $KH_2PO_4$, 2.7 mM KCl, and 137 mM NaCl, pH 7.4) in the presence of 0.1 µM 1.12.2 aptamer, 0.93 nM RQDs and 0.12 mg/mL amSWCNTs.

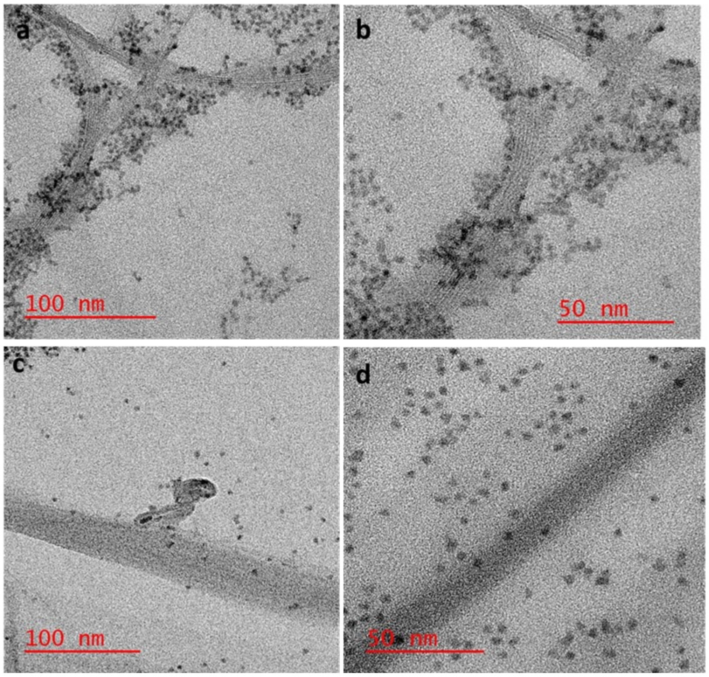


Fig. S7. HR-TEM images of nanocomplex **1** are the mixture of A08min-mQDs and amSWCNT, where the QDs are closely associating with the amSWCNT side wall (a and b). Nanocomplex **1** after the addition of 1.2 µM of OTA, OTA specific binding with its aptamer and resulting in disassembly of the QDs (c and d). Nanocomplex **1** assembly and disassembly were found throughout the sample area in the grid and the images are from many different regions.

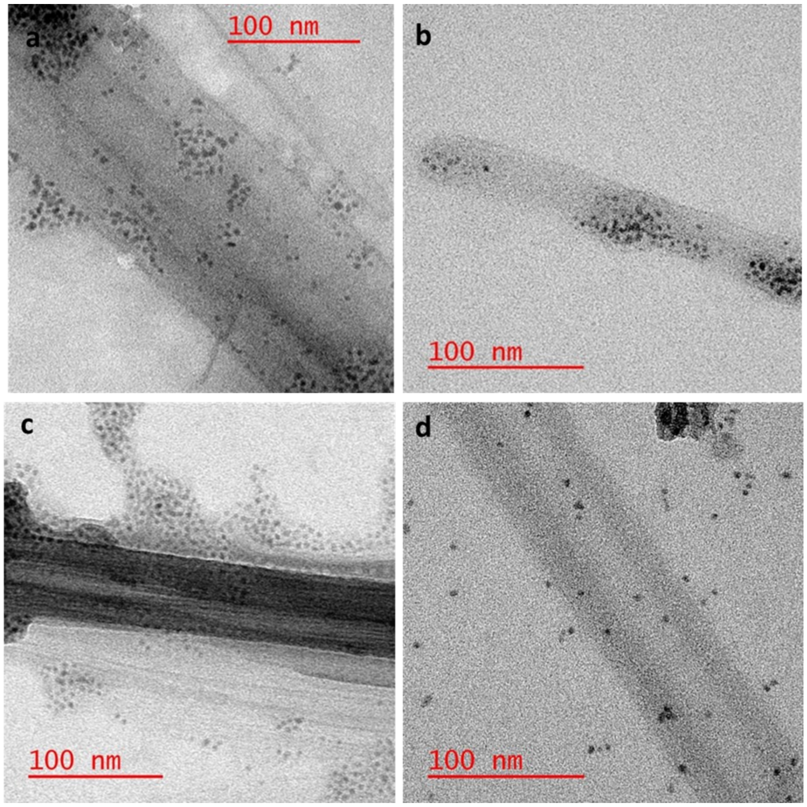


Fig. S8. HR-TEM images of nanocomplex **2** are the mixture of A08min-mQDs and SWCNT, where the QDs are closely associating with the SWCNT side wall (a and b). Nanocomplex **2** after the addition of 1.2 μM of OTA, OTA specific binding with its aptamer and resulting in disassembly of the QDs (c and d). Nanocomplex **2** assembly and disassembly were found throughout the sample area in the grid and the images are from many different regions.

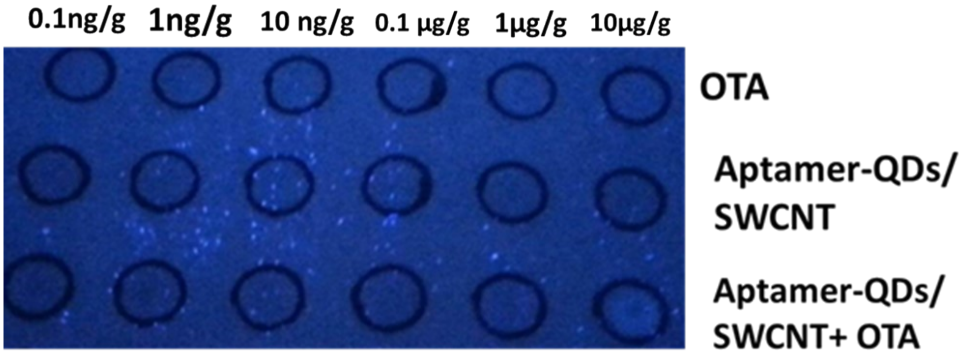


Fig. S9. Paper-based nanocomplex **2** sample (1 mL) was spotted into the bottom two rows of black circles. Top: 1 μL OTA from$10^{-4}$ to $10^{-9}$ M spiked into complex extract as a control. Middle: 1 μL of complex extract spotted onto each of the nanocomplex **2** zones. Bottom: 1μL OTA from$10^{-4}$ to $10^{-9}$ M spiked into complex extract and spotted on the nanocomplex **2** zones.

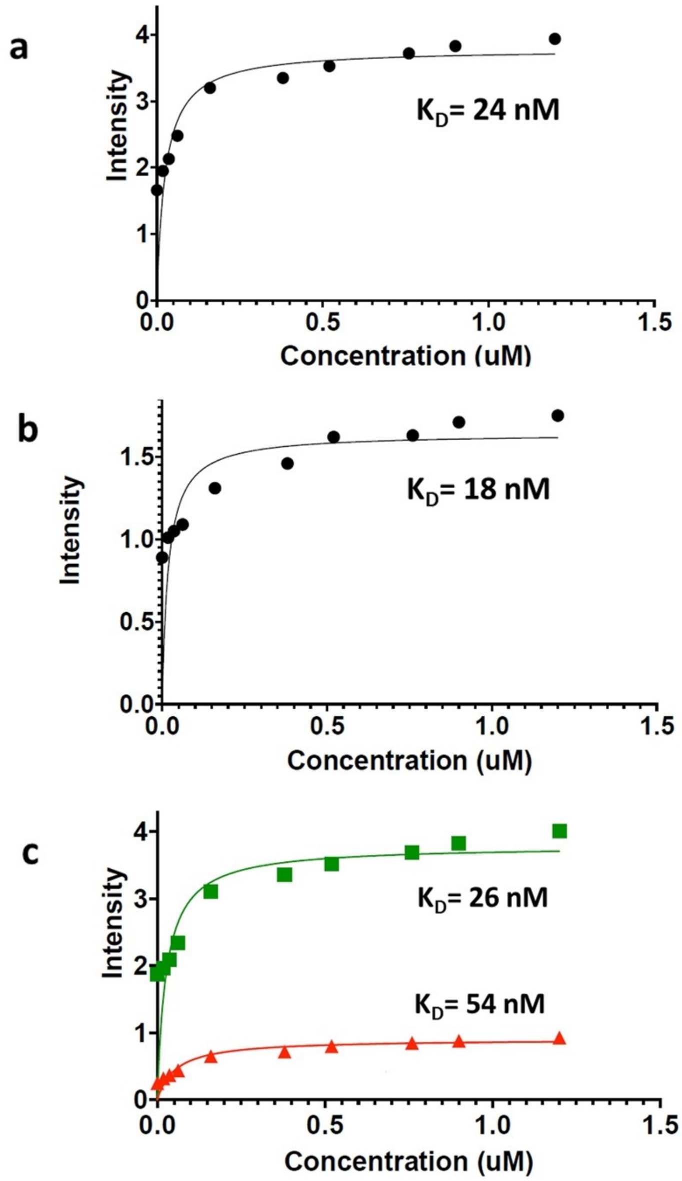


Fig. S10. The apparent $K_D$ values were determined for the A08min aptamer obtained using the fluorescence experimental data through nonlinear regression analysis by fitting the data with the one site specific binding equation using GraphPad Prism 6 software. A). Nanocomplex **3** (using

A08min-GQD) with OTA, B). Nanocomplex **4** (using A08min-RQD) with OTA, and C). Nanocomplex **1** (using A08min-both GQD and RQD) with OTA
